# DNA barcoding of Notopterygii Rhizoma et Radix (Qiang-huo) and identification of adulteration in its medicinal services

**DOI:** 10.1038/s41598-024-53008-0

**Published:** 2024-02-04

**Authors:** Zhen-Wen Liu, Jing Zhou

**Affiliations:** 1 Yunnan Key Laboratory of Biodiversity of Gaoligong Mountain, Yunnan Academy of Forestry and Grassland, Kunming, 650201 China; 2 Gaoligong Mountain, Forest Ecosystem, Observation and Research Station of Yunnan Province, Kunming, 650201 China; 3 School of Pharmaceutical Science and Yunnan Key Laboratory of Pharmacology for Natural Products, Kunming Medical University, 1168 Western Chunrong Road, Yuhua Street, Chenggong New City, Kunming, 650500 China

**Keywords:** Plant genetics, Plant molecular biology

## Abstract

Safety concerns, stemming from the presence of complex and unpredictable adulterants, permeate the entire industrial chain of traditional Chinese medicines (TCMs). The Notopterygii Rhizoma et Radix (NReR) from the Apiaceae family, commonly known as “Qiang-huo”, is a widely used herbal medicine. The recent surge in its demand has given rise to a proliferation of counterfeit and substituted products in the market. Traditional identification presents inherent limitations, while DNA mini-barcoding, reliant on sequencing a short-standardized region, has received considerable attention as a new potential means to identify processed medicinal materials. In this study, we constructed a comprehensive Internal Transcribed Spacer 2 (ITS2) matrix encompassing genuine NReR and their commonly found adulterants for the first time. Leveraging this matrix, we conducted a thorough assessment of the genetic profiles and sources of NReR available in the Chinese herbal medicine market. Following established DNA barcoding protocols, the intra-specific genetic divergences within NReR species were found to be lower than the inter-specific genetic divergences from other species. Among the 120 samples that were successfully amplified, ITS2 exhibits an outstanding species-level identification efficiency of 100% when evaluated using both the BLASTN and neighbor-joining (NJ) tree methods. We concluded that ITS2 is a mini-barcode that has shown its potential and may become a universal mini-barcode for the quality control of “Qiang-huo”, thereby ensuring the safety of clinical medication.

## Introduction

Traditional Chinese medicine (TCM), encompassing Chinese herbal medicine, continues to gain international recognition. According to the National Bureau of Statistics of China, the turnover of the Chinese herbal medicine market in 2019 reached 165.3 billion yuan for the domestic market and $6.175 billion for the international side^1^. Apiaceae, a family of flowering plants, is recognized as a significant resource for TCM, comprising 65 genera and 262 species^2^. Among them, Notopterygii Rhizoma et Radix (NReR), known as "Qiang-huo" in Chinese, holds a long history dating back to the Han Dynasty, approximately 2000 years ago^3^. According to the Pharmacopoeia of the People's Republic of China, NReR is derived from the roots and rhizomes of *Notopterygium incisum* Ting ex H. T. Chang or *N. franchetii* H. de Boissieu^4^. It encompasses a complex array of chemical constituents, including volatile oils and terpenes, coumarins, sugars, glycosides, phenolic acids, polyalkynes, and alkaloids^5^. Modern pharmacological studies have demonstrated its anti-inflammatory, antibacterial, antioxidant, antiarrhythmic, anticancer, antipyretic, and analgesic activities^6^. Currently, NReR serves as a raw material for over two hundred types of Chinese (Tibetan) patent medicines.

*Notopterygium incisum* and *N. franchetii* were listed as national third-class protected plants as early as 1987, and they were successively included as "Near-Threatened" species in China's Red List of Biodiversity and China Species Red List. In the past few decades, excessive excavation and habitat destruction lead the wild resources of NReR to be drastically reduced^7–9^. The scarcity of resources and the increase of market demand have driven the price up, which motivated adulteration intentionally. Reports indicate that *Angelica sylvestris* L., *Pleurospermum rivulorum* (Diels) Hiroe, *Polygonum cuspidatum* Sieb et Zucc, and *Sanguisorba officinalis* L. are frequently sold as NReR in the medicinal market^10–12^. These species share similar organoleptic characteristics but differ in chemical constituents compared to NReR^13–17^. Traditional methods used to authenticate NReR and its adulterants, such as macroscopy^11,18,19^, microscopy^20^, and chemical profiles^21,22^, can provide certain recognition and differentiation to some extent. But, these methods are prone to geographical variations, growth stages, and storage conditions, which may affect identification accuracy^23^. Therefore, it is necessary to establish a simple and accurate identification method to distinguish NReR from adulterations.

DNA barcode technology is currently used as an effective tool to identify species. This method provides a large amount of genetic information with high accuracy and objectivity, and it can standardize and automate the identification process, establishing an easy-to-use application system in a short time^23^. One potential DNA barcode for identifying medicinal plants and their close relatives is the internal transcribed spacer 2 (ITS2), which has attracted attention due to its unique advantages such as being short and conducive to amplifying degraded samples^24,25^. While a few studies have examined the molecular identification of NReR and its adulterants using DNA barcoding^26–28^, the composition of commercial NReR in the Chinese medicinal market needs to be sorted out to ensure the subsequent formulation of quality control standards and clinic safety for NReR.

Thus, the aim of this study is to investigate whether ITS2 is a valuable marker for identifying genuine NReR from its adulterants and to gain insight into the composition of commercial NReR in China.

## Materials and methods

### Plant materials

Eighteen ITS sequences available for genuine NReR (*N. incisum* and *N. franchetii*) were first downloaded and screened from NCBI GenBank as reference. Moreover, 168 commercial crude drug samples under the name of NReR were collected from herbal markets, pharmacies, and online shops in 23 provinces and municipalities of China. Voucher specimens are deposited in Herbarium of Kunming Medical University. Detailed information is presented in the Table S1. All methods of experimental research on plants were performed in accordance with the relevant institutional, national, and international guidelines and legislation.

### DNA extraction, polymerase chain reaction (PCR) amplification, and sequencing

The surface of all herbal materials was cleaned with 75% ethanol to avoid fungal DNA contamination. About 50 mg of the materials were cut into pieces, added with 10% polyvinylpyrrolidone (PVP), and then ground with a FastPrep bead mill (Retsch MM400, Germany). Total genomic DNA was extracted using the modified CTAB procedure of Doyle and Doyle^29^ or using the Plant Genomic DNA Kit (Tiangen Biotech, Beijing, China). Agarose gel electrophoresis showed slight smearing in some DNA samples, indicating partial degradation. The universal primers ITS-S2F (5′-ATGCGATACTTGGTGTGAAT-3′) and ITS-S3R (5′-GACGCTTCTCCAGACTACAAT-3′) were used to amplify the complete ITS2 region^24^. The PCR reaction conditions were the same as described previously^30^. PCR products purifying and sequencing were completed by Tsingke Biotechnology Co., Ltd (Beijing, China).

### Data analysis

The sequences of genuine and commercial NReR were assembled using the MAFFT v.7^31^, and manually adjusted where necessary using the BioEdit^32^. The assembled sequences were annotated and trimmed to obtain the complete ITS2 region based on a Hidden Markov Model (HMM)^33^. The genetic distances were calculated using MEGA v.7^34^ according to Kimura 2-parameter (K2P) model^34^. Barcoding gaps comparing the distributions of the pairwise intra- and inter-specific distances with distance intervals of 0.002 were estimated in Microsoft Excel 2016. The true NReR presenting a minimum inter-specific distance value higher than their maximum intra-specific distance were considered successfully discriminated from potential adulterant plant species^35^. Wilcoxon two-sample tests were performed as described previously^24,36^. Haplotype matrix was generated by DNAsp v.6^37^. BLASTN and the nearest distance methods were both used to evaluate the species authentication efficacy^38^. Sequences were uploaded onto NCBI database with a minimum identity cut of 99% for a top match according to the BLAST program (http://blast.ncbi.nlm.nih.gov/Blast.cgi). Neighbor-joining (NJ) tree was constructed based on haplotypes, performing 1,000 bootstrap replicates in MEGA v.7^34^.

## Results

### Amplification, sequencing and sequence characteristics

Genomic DNA was extracted from a total of 168 commercial "Qiang-huo" products, out of which 48 samples failed to amplify due to severe DNA degradation. The length of 138 combined sequences ranges from 226 to 256 bp. The GC content of the sequences shows a mean value of 58.3% with a range of 53.2% to 68.2%. The aligned length of 263 bp exhibits 143 variable sites, a rate of 54.4% (Table 1). These findings suggest that sequences for the sampled "Qiang-huo" were relatively variable.

**Table 1:** ITS2 sequence characters of samples.

DNA extraction efficiency (%)	71.4
Amplification efficiency (%)	100%
Length of all taxa (bp)	226–256
Aligned length (bp)	263
G + C content range in all taxa (%)	53.2–68.2
Number (and %) of variable sites in all taxa	143 (54.4%)

### Assessment of barcoding gap

**Figure 1:**
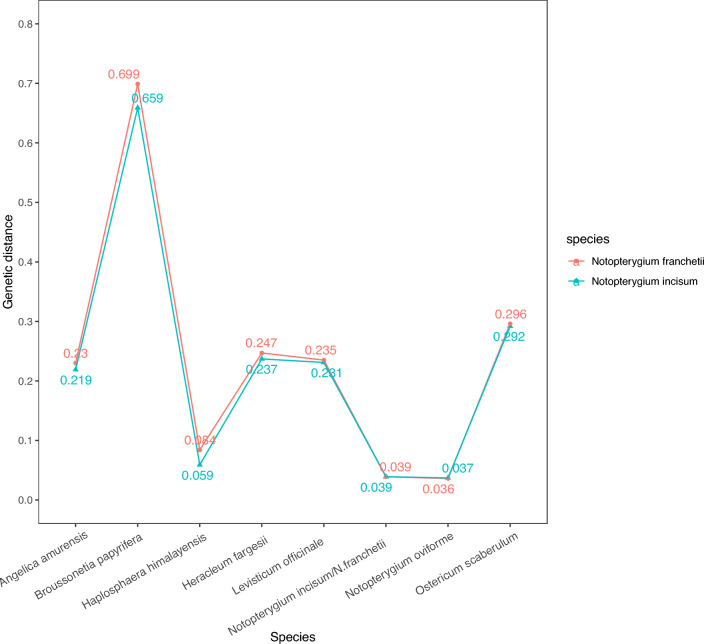
Genetic distances from genuine NReR to its adulterants.

**Figure 2:**
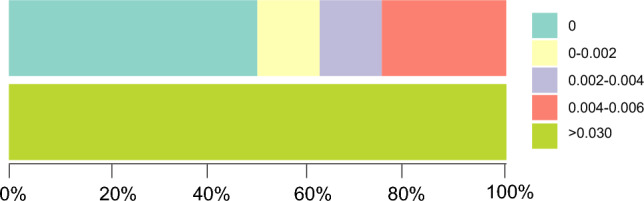
Relative distribution of inter-specific divergences and intra-specific variations for ITS2 sequences. The colored bars in each box represent inter-specific (above) and intra-specific (below) genetic distances.

The average interspecific distance between *N. incisum* and *N. franchetii* was 0.039. The interspecific distance between *N. incisum* and the adulterant species ranges from 0.037 (*N. oviforme*) to 0.659 (*Broussonetia papyrifera*). *Notopterygium franchetii* shows a similar interspecific distance with the adulterants, with the maximum interspecific distance being 0.699 from *B. papyrifera* and the minimum being 0.036 from *N. oviforme.* The intraspecific genetic distance within *N. incisum* (0.005) and *N. franchetii* (0.005) was both smaller than interspecific distance between NReR and adulterants (Fig. 1). Our results show that the intra- and inter-specific variation of ITS2 had distinct gaps (Fig. 2). Additionally, Wilcoxon’s two-sample tests reveals that the mean of the inter-specific divergences was significantly higher than that of the corresponding intra-specific variations (*p* < 0.001, Table 2).

**Table 2:** Wilcoxon two-sample tests for distribution of intra- vs. inter-specific divergences.

No. of inter-specific distances	No. of intra-specific distances	Wilcoxon W	*P* value
36	9	288	1.251e-05

### Evaluation of species authentication capability of ITS2

The BLASTN method exhibits a 100% success rate in identifying the tested commercial samples (Table 3). These samples consist of nine species, namely *N. incisum*, *N. franchetii*, *N. oviforme*, *Levisticum officinale*, *A. amurensis*, *Ostericum scaberulum*, *B. papyrifera*, *Haplosphaera himalayensis*, and *Heracleum fargesii*. Each identification result was supported by best hit of accessions obtained from the NCBI database (Table S1). A few samples initially identified as *O. scaberulum*, *B. papyrifera*, *Ha. himalayensis* and *He. fargesii* were subsequently confirmed through additional sampling and sequencing.

**Table 3:** Comparison of authentication efficiency for ITS2 using different methods.

Methods of identification	No. of samples	No. of species	Correct identification (%)	Incorrect identification (%)	Ambiguous identification (%)
BLASTN	120	9	100	0	0
Distance	120	9	100	0	0

**Figure 3:**
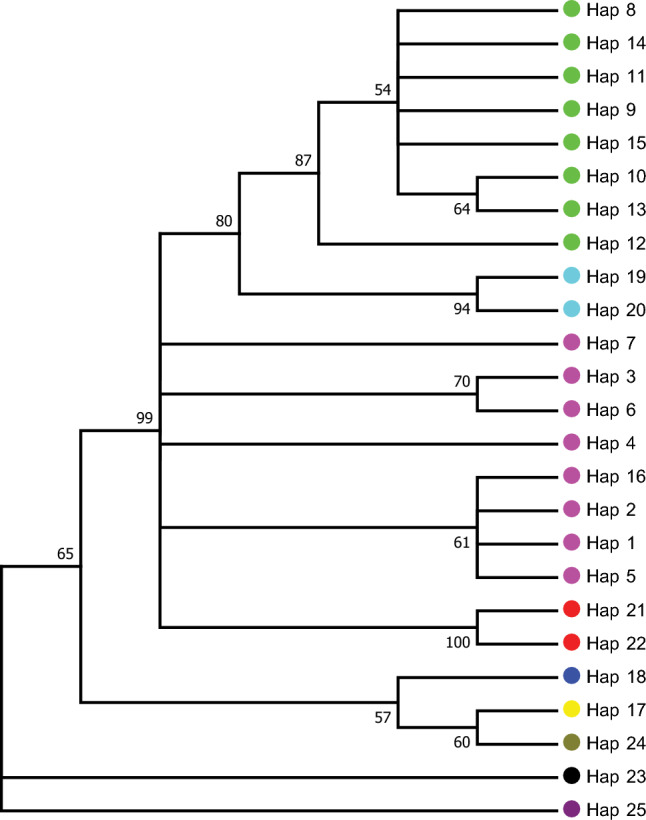
Neighbor joining (NJ) tree of NReR and its adulterants constructed based on the haplotypes. Detailed information about the haplotypes associated with each species is shown in Table 4. Bootstrap values are shown (≥ 50%) next to the branch.

A total of 25 haplotypes were generated from the ITS2 sequences of genuine NReR and 120 commercial samples (Fig. S1). Combining these haplotypes with the BLASTN results, *N. incisum* was assigned to Haps_1-7, *N. franchetii* to Haps_8-15, *L. officinale* to Hap_17, *A. amurensis* to Hap_18, *O. scaberulum* to Haps_19-20, *Ha. himalayensis* to Haps_21-22, *O. scaberulum* to Hap_23, *He. fargesii* to Hap_24, and *B. papyrifera* to Hap_25 (Table 4). With the exception of *N. incisum*, the NJ tree analysis reveals that haplotypes representing different species formed isolated clades. While the haplotypes representing *N. incisum* does not form a monophyletic group on the NJ tree, the sequences to be identified as potential authentic species were clustered together with haplotypes representing genuine species (Fig. 3). Hence, the NJ tree method also exhibits a 100% success rate for NReR identification (Table 3).

**Table 4:** Information on the haplotypes associated with commercial NReR samples. The species identity of the samples was determined by BLAST queries on NCBI. Sequences of the joining (NJ) tree of NReR and its adulterantsgenuine NReR downloaded and screened from GenBank are highlighted in bold.

Haplotype	Number	Sample
Hap_1	23	***Notopterygium incisum*_MF096520**, ***N. incisum*_MF787530**, ***N. incisum*_MF787529**, *N. incisum*_HB2, *N. incisum*_HB3, *N. incisum*_HB5, *N. incisum*_HB8, *N. incisum*_HB9, *N. incisum*_GZ2, *N. incisum*_GZ4, *N. incisum*_JL1, *N. incisum*_QH1, *N. incisum*_SC3, *N. incisum*_SC4, *N. incisum*_SC5, *N. incisum*_SC6, *N. incisum*_SC8, *N. incisum*_YN5, *N. incisum*_YN11, *N. incisum*_GX5, *N. incisum*_GX8, *N. incisum*_SX2, *N. incisum*_SD4
Hap_2	1	***N. incisum*_MF787525**
Hap_3	1	***N. incisum*_MF787523**
Hap_4	24	***N. incisum*_EU236180**, *N. incisum*_HLJ4, *N. incisum*_HLJ3, *N. incisum*_HLJ10, *N. incisum*_GS5, *N. incisum*_GS10, *N. incisum*_QC7, *N. incisum*_SC9, *N. incisum*_SX1, *N. incisum*_SX6, *N. incisum*_SX8, *N. incisum*_GX1, *N. incisum*_GD1, *N. incisum*_GD2, *N. incisum*_GD5, *N. incisum*_AH6, *N. incisum*_AH11, *N. incisum*_SC2, *N. incisum*_JS1, *N. incisum*_YN2, *N. incisum*_YN12, *N. incisum*_ZJ1, *N. incisum*_ZJ2, *N. incisum*_GD7
Hap_5	1	***N. incisum*_MF787528**
Hap_6	2	***N. incisum*_MF787518**, *N. incisum*_AH9
Hap_7	1	***N. incisum*_JQ936558**
Hap_8	1	***N. franchetii*_MF787573**
Hap_9	21	***N. franchetii*_MH807979**, ***N. franchetii*_KX674898**, *N. franchetii*_GD4, *N. franchetii*_GZ1, *N. franchetii*_AH2, *N. franchetii*_AH3, *N. franchetii*_AH7, *N. franchetii*_AH8, *N. franchetii*_HB4, *N. franchetii*_HB6, *N. franchetii*_JX1, *N. franchetii*_SD5, *N. franchetii*_YN4, *N. franchetii*_GD8, *N. franchetii*_HN1, *N. franchetii*_SX4, *N. franchetii*_QC6, *N. franchetii*_AH22, *N. franchetii*_GS8, *N. franchetii*_GS4, *N. franchetii*_SX7
Hap_10	1	***N. franchetii*_MN049518**
Hap_11	1	***N. franchetii*_MF787569**
Hap_12	1	***N. franchetii*_MF787568**
Hap_13	1	***N. franchetii*_MF787578**
Hap_14	1	***N. franchetii*_MF096527**
Hap_15	2	***N. franchetii*_KX675119**, *N. franchetii*_HB7
Hap_16	1	*N. incisum*_GS1
Hap_17	17	*Levisticum officinale*_AH15, *L. officinale*_AH18, *L. officinale*_AH20, *L. officinale*_AH26, *L. officinale*_GS3, *L. officinale*_CQ1, *L. officinale*_CQ11, *L. officinale*_GX6, *L. officinale*_HLJ7, *L. officinale*_HJL9, *L. officinale*_LL1, *L. officinale*_LN1, *L. officinale*_LN2, *L. officinale*_LN3, *L. officinale*_LN6, *L. officinale*_SX5, *L. officinale*_SC13
Hap_18	13	*Angelica amurensis*_AH4, *A. amurensis*_AH16, *A. amurensis*_AH17, *A. amurensis*_AH23, *A. amurensis*_AH24, *A. amurensis*_AH25, *A. amurensis*_CQ12, *A. amurensis*_GS2, *A. amurensis*_NM1, *A. amurensis*_SD2, *A. amurensis*_GD3, *A. amurensis*_AH13, *A. amurensis*_GS7
Hap_19	13	*N. oviforme*_AH5, N. oviforme_AH10, *N. oviforme*_AH12, *N. oviforme*_AH14, *N. oviforme*_AH19, *N. oviforme*_SC12, *N. oviforme*_SC15, *N. oviforme*_SD1, *N. oviforme*_SD3, *N. oviforme*_JX2, *N. oviforme*_JL2, *N. oviforme*_HB1, *N. oviforme*_QC5
Hap_20	2	*N. oviforme*_YN9, *N. oviforme*_YN10
Hap_21	1	*Haplosphaera himalayensis*_XZ1
Hap_22	1	*Ha. himalayensis*_XZ2
Hap_23	5	*Ostericum scaberulum*_YN1, *O. scaberulum*_YN6, *O. scaberulum*_YN7, *O. scaberulum*_YN8, *O. scaberulum*_GX3
Hap_24	1	*Heracleum fargesii*_SC16
Hap_25	2	*Broussonetia papyrifera*_YN13, *B. papyrifera*_YN14

### Survey of commercial NReR in the Chinese medicine markets

**Figure 4:**
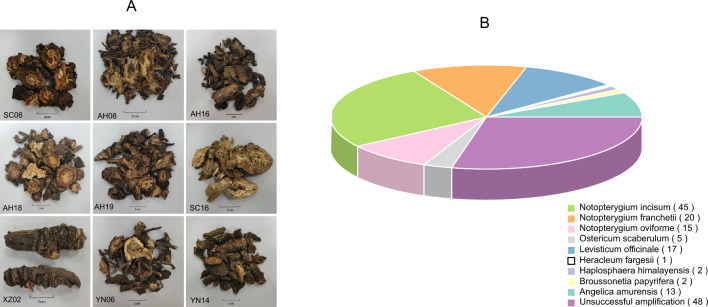
(**A**) Morphology of commercial “Qiang-huo” (SC06: *Notopterygium incisum*, AH08: *N. franchetii*, AH16: *Angelica amurensis*, AH18: *Levisticum officinale*, AH19: *N. oviforme*, SC16: *Heracleum fargesii*, XZ02: *Haplosphaera himalayensis*, YN06: *Ostericum scaberulum*, YN14: *Broussonetia papyrifera*), (**B**) The composition and proportion of the commercial “Qiang-huo” products identified by barcode ITS2.

This study represents the most comprehensive nationwide sampling of commercial “Qiang-huo” to date, comprising a total of 168 samples obtained from 23 provinces. Based on their external morphology and odor characteristics, these samples proved challenging to distinguish from one another (Fig. 4A). Of 168 samples, except 48 failed to be amplified, molecular identification results show that 65 samples (54.2%) were identified as authentic “Qiang-huo”, while 55 samples (45.8%) were identified as adulterants (Fig. 4B). Further identification using the BLASTN method reveals that the adulterants belonged to seven different species, namely *L. officinale* (17 samples), *A. amurensis* (13 samples), *O. scaberulum* (five samples), *B. papyrifera* (L.) Vent. (two samples), *Ha. himalayensis* (two samples) and *He. fargesii* (one sample) (Table S1). *Levisticum officinale*, the most widely sold adulterant, was found in medicinal markets of nine provinces, followed by *N. oviforme*, *A. amurensis* and *O. scaberulum* found in eight, six and two provinces, respectively. Notably, *B. papyrifera* and *Ha. himalayensis* were only detected in Yunnan and Xizang, respectively. Regarding spatial distribution, samples from Guizhou, Hunan, Jiangsu, Qinghai, and Zhejiang were all confirmed as genuine NReR, while in Chongqing, Liaoning, Inner Mongolia, Shaanxi, and Xizang, no authentic NReR was detected. In ten provinces, including Hubei, Jilin, Jiangxi, and others, only one adulterant was discovered. Similarly, within the six provinces, such as Sichuan, Guangxi, Gansu, and others, two distinct types of adulterants were observed. The scenario in Yunnan and Anhui is more complicated, as three distinct types of adulterants were found (Tables S1, S2).

## Discussion

In recent years, DNA molecular identification technology has emerged as a robust tool for TCMs identification. This technology stands out for its ease of operation, cost-effectiveness, and high accuracy. In 2009 at the 3rd World DNA Barcode Conference, it was announced that the *mat*K and *rbc*L markers are the core sequences of plant DNA barcodes, with ITS and *trn*H-*psb*A as complementary sequences^39^. The longer length of these markers has shown some weakness in amplification, sequencing and alignment, which is exacerbated if the materials are highly processed and have degraded DNAs^40,41^. Unlike Western herbs, most Chinese medicinal herbs are subjected to traditional processing procedures to increase their potency, minimize negative effects, and change their medicinal properties for a particular clinical use before they are released into dispensaries, practitioners, and the market^42^. According to the Chinese pharmacopoeia, the medicinal herbs are typically cleaned, cut, dried, and then processed, including stir-frying, charring, steaming, boiling, and calcining^4,43^. Raw materials processing methods can cause DNA degradation, posing challenges in obtaining standard DNA barcode sequences for samples. The use of mini-DNA barcoding technology, which focuses on shorter yet more efficiently amplified sequences through PCR, can partially overcome this limitation^24,41,44,45^.

ITS2, a mini-barcode spanning 160 to 330 bp in length, has emerged as the predominant marker for identifying plant medicinal materials^24^. Its growing popularity can be attributed to its ease of amplification and remarkable discriminatory capabilities across different taxonomic levels^24^. In this study, ITS2 performs well, with a higher amplification rate of 71.4%. Unsuccessfully PCR amplified NReR samples could be attributed to the high temperature drying process, causing DNA degradation. Both nucleotide signature (NS) and genome skimming metagenomics (GSM) emerge as promising solutions for fragmented and degraded plant materials identification^46^. NS consists of distinct nucleotide sequences that are exclusive to a specific taxonomic group. Previous studies, such as those on American Ginseng^47^, Cistanches Herba^48^, and Pinelliae Rhizoma^49^, have successfully demonstrated the efficacy of nucleotide signatures in identifying medicinal materials. GSM is the low-coverage shotgun sequencing of total DNA. When this approach is applied on herbal products, sequencing library is built without PCR amplification of barcode regions, circumventing the limitations of PCR in conventional DNA barcoding, such as DNA degradation during product manufacturing and PCR bias because of primer mismatch, etc. GSM produces millions of reads in a single run. After quality control, reads could then be clustered into operational taxonomic units (OUTs) based on similarity at defined threshold (usually 99–100%). Representative consensus sequences from each cluster would then be subject to taxonomic assignment, usually by alignment-based software like BLAST or k-mer based methods like Kraken^46,50^. As its sequencing cost is decreasing year by year, GSM technology will be a prospective method for the identification of TCMs herbs.

The validity of DNA barcoding relies heavily on the availability of a precise reference database, as it serves as the cornerstone of DNA barcoding. In this study, we constructed a comprehensive ITS2 matrix encompassing genuine NReR and their commonly found adulterants for the first time. This matrix will play a crucial role in both monitoring NReR and exploring potential substitute sources. The matrix includes two authentic species of NReR (*N. incisum* and *N. franchetii*, comprising 16 haplotypes) and seven confused species (nine haplotypes) (Table 4; Fig. S1). Out of the 120 samples sold as "Qiang-huo", only 54.2% were identified as authentic NReR, while the rest were identified as adulterants, including *L. officinale*, *N. oviforme*, *A. amurensis*, *O. scaberulum*, *He. fargesii*, *Ha. himalayensis*, and *B. papyrifera* (Fig. 4B). These findings highlight the complexity of the "Qiang-huo" market, with the presence of previously unreported species. According to the Chinese Pharmacopeia, only *N. incisum* and *N. franchetii* are listed as sources of NReR, and the former one exhibits superior quality and efficacy^51^. Our analysis revealed that *N. incisum* accounted for two-thirds of the authentic products, further indicating a preference for *N. incisum* in the market (Fig. 4B; Table S1). The NJ tree showed that adulterants of NReR were distantly related to *N. incisum* and *N. franchetii* (Fig. 3). *Levisticum officinale* was introduced to China in 1957, and used as a substitute for the traditional Chinese medicine “dang gui”, the roots of *A. amurensis*, *He. fargesii* and *O. scaberulum*^52^. The chemical and pharmacological analysis results of *L. officinale* and *A. amurensis* dramatically differs from those of NReR^53–58^. *Notopterygium oviforme* and *Ha. himalayensis* were grouped together in a strongly supported clade (bootstrap support = 99), closely related to authentic NReR, exhibiting genetic distances of 0.059–0.084 and 0.036–0.037, respectively (Figs. 1, 3). While *N. oviforme* has been traditionally regarded as a regional substitute to NReR, albeit with inferior quality^59^, additional research is urgently needed to explore the chemical constituents and pharmacological efficacy of both *N. oviforme* and *Ha. himalayensis*. This investigation aims to ascertain their potential as viable substitutes for NReR. *Broussonetia papyrifera* may represent a contaminant in the Yunnan samples, as its morphology significantly differs from that of NReR, and we also detected genuine NReR in these samples.

## Conclusions

In this study, the origin plants of commercial “Qiang-huo” in the market was clarified, and the reference matrix of NReR and its adulterants was successfully established. This achievement is crucial for the future industrial development of NReR. However, it is important to acknowledge that the amplification efficiency of the ITS2 region is not always optimal, which is a challenge encountered in DNA barcoding identification of many medicinal materials. Therefore, alternative approaches such as NS and GSM appear to be promising solutions for overcoming this issue. These cutting-edge methodologies have the potential to revolutionize the field, providing more comprehensive and accurate identification results for medicinal materials.

### Supplementary Information

Supplementary Figure S1.

Supplementary Tables.

## Data Availability

New sequenced and other published ITS2 sequences can be found in GenBank (https://www.ncbi.nlm.nih.gov/genbank/), and the accession numbers showed in Table S1.
