# Supplementary material for: DNA barcoding of Notopterygii Rhizoma et Radix (Qiang-huo) and identification of adulteration in its medicinal services
Source: Sci Rep. 2024 Feb 4;14:2879. doi: 10.1038/s41598-024-53008-0 (PMC10838912; doi:10.1038/s41598-024-53008-0)
Supplement: Supplementary file 1 — Supplementary Figure S1. [file 41598_2024_53008_MOESM1_ESM.pdf]

Figure S1. The ITS2 haplotypes matrix of commercial NReR.

```
[               10        20        30        40        50        60        70        80        90       100       110       120       130     ]
[                *         *         *         *         *         *         *         *         *         *         *         *         *     ]
Hap_1   GTCGTGTTGCTGCTTCTCCTGAGGAGATAGCCTCTGTACGTGTTGCGGCGTGCCCGCTCTCGCGCTACTTTGAAGACCCTATCTCTTTGCGCAAACCCGTACCAAGGGAGTCGATTTCTCGCCAAACTCGCT
Hap_2   .............................C........................................................................................................
Hap_3   ..............................................T..........................................C............................................
Hap_4   .........................................................................................C............................................
Hap_5   .........................A..................................................A.....................................A...................
Hap_6   ...............T..............................T..........................................C............................................
Hap_7   ...............................................................T.........................C......................................T.....
Hap_8   ..............................A.G.....T...........CT..................................T..C..A...............C...................T.....
Hap_9   ................................G.....T...........CT..................................T..C..A...............C...................T.....
Hap_10  ................................G.....T...........CT..................................T..C..A...............C....T..............T.....
Hap_11  ................................G.....T...........CT........................C.........T..C..A...............C...................T.....
Hap_12  ................................G.....T...........CT..................................T..C..A...............T...................T.....
Hap_13  ............................T...G.....T...........CT..................................T..C..A...............C....T..............T.....
Hap_14  ..............................A.G.....T...........CT........................C.........T..C..A...............C...................T.....
Hap_15  .........T......................G.....T...........CT................................G.T..C..A...............C...................T.....
Hap_16  ........C.............................................................................................................................
Hap_17  ...A.C....AA..A...........C.T..TGT..ATT......TT.T.C.TG..T.TC..T.T....T..GT........CAG....C......C.G....T.T..T.....T.G..AGT.G..G....T...
Hap_18  .....A....AA..A.A.A....A..T.T....T..A.T..A...TC.T.C.TGGA...C..T....C...CG.........C.G....C......C.T....T.T..C.....A.G..AG.AG..T.......T.
Hap_19  ..............................T.................T.C...........................A..........A..A...............C...................T.....
Hap_20  ..............................T.......T.........T.C...........................A..........A..A...............C...................T.....
Hap_21  ........A...T......................A..............C...T.....T...T.G................A.....C...T..............A.....................T.T.
Hap_22  ........A...T......................A..............C...T.....T.....G................A.....C...T..............A.....................T.T.
Hap_23  ...A......A..AA..T.CTT...T..TA...TCT..T....A.TT.T.C.TG.ATAT.T....ATC...CG.......T.C.G....C...T..GT..T....T..TTG.....G..AG....TCCT.T.T.
Hap_24  ..TCAC....AA.AA...........C.C.TT.T..A.TA....CTC..A..TAA.T....TT.........G.........C.G....C.G....C.GG...T.T.AC.....C.G..AG..G..C.T.....
Hap_25  ACG..TGCC.CA.CA.C.ACA.AAC.CCT...CTC.CGA.C.C...CT....T..TTCTCTC.T.A..G.CCG.CGTC.TACGGGC.CCCA..TCGTGTTCGCTGCG..ACTCTACACGT....A.GCTG.CAC
```
