# Supplementary material for: DNA barcoding of Notopterygii Rhizoma et Radix (Qiang-huo) and identification of adulteration in its medicinal services
Source: Sci Rep. 2024 Feb 4;14:2879. doi: 10.1038/s41598-024-53008-0 (PMC10838912; doi:10.1038/s41598-024-53008-0)
Supplement: Supplementary file 2 — Supplementary Tables. [file 41598_2024_53008_MOESM2_ESM.docx]

| **Table S1.** The BLAST results of the samples sold as “Qiang-huo” from different medicinal markets in China | | | | | | |
| --- | --- | --- | --- | --- | --- | --- |
| Sample no. | Species identified by BLAST | Best-hit of representative accessions | Identity | Test results | Province | GenBank  accession No. |
| AH01 | Unsuccessful amplification | / | / | / | Anhui | / |
| AH02 | *Notopterygium franchetii* | MF096526; MF787580; KY848846; KX674897; JQ936555; MH807979; JF755943; JF694085. | 100 | G | Anhui | OR030164 |
| AH03 | *Notopterygium franchetii* | MF096522; MF787580; KY848846; KX674897; JQ936555; MH807979; JF755943; JF694085. | 100 | G | Anhui | OR030165 |
| AH04 | *Angelica amurensis* | MH188440; MW433735; MN877395; MT735426; JN603227; GU395148; DQ263581. | 100 | A | Anhui | OR030243 |
| AH05 | *Notopterygium oviforme* | MF787548; KY848837; MT337430. | 100 | A | Anhui | OR030257 |
| AH06 | *Notopterygium incisum* | MF096521; MF787531; KY848836; KX674900; MN049521; MT337434; JQ936557; KJ999463; GQ379335; EU236180. | 100 | G | Anhui | OR030217 |
| AH07 | *Notopterygium franchetii* | MF096526; KY848846; KX674897; JQ936555; MH807979; JF755943; JF694085. | 100 | G | Anhui | OR030166 |
| AH08 | *Notopterygium franchetii* | MF096526; MF787580; KY848846; KX674897; JQ936555; MH807979; JF755943; JF694085. | 100 | G | Anhui | OR030167 |
| AH09 | *Notopterygium incisum* | MF787518; MF787516. | 100 | G | Anhui | OR030256 |
| AH10 | *Notopterygium oviforme* | MF787548; KY848837; MT337430. | 100 | A | Anhui | OR030258 |
| AH11 | *Notopterygium incisum* | MF096521; MF787531; KY848836; KX674900; MN049521; MT337434; JQ936557; KJ999463; GQ379335; EU236180. | 100 | G | Anhui | OR030218 |
| AH12 | *Notopterygium oviforme* | MF787548; KY848837; MT337430. | 100 | A | Anhui | OR030259 |
| AH13 | *Angelica amurensis* | MH188440; MW433735; MN877395; MT735424; JN603227; GU395148; DQ263581. | 100 | A | Anhui | OR030254 |
| AH14 | *Notopterygium oviforme* | MF787548; KY848837; MT337430. | 100 | A | Anhui | OR030260 |
| AH15 | *Levisticum officinale* | MG218304; MG770573; KT944674; KC812809; GU395146. | 100 | A | Anhui | OR030226 |
| AH16 | *Angelica amurensis* | MH188440; MW433735; MN877395; MT735426; MT735425; JN603227; GU395148; DQ263581. | 100 | A | Anhui | OR030244 |
| AH17 | *Angelica amurensis* | MH188440; MW433735; MN877395; MT735426; JN603227; GU395148; DQ263581. | 100 | A | Anhui | OR030245 |
| AH18 | *Levisticum officinale* | MG218304; MG770573; KT944674; KC812809; GU395146. | 100 | A | Anhui | OR030227 |
| AH19 | *Notopterygium oviforme* | MF787548; KY848837; MT337430. | 100 | A | Anhui | OR030261 |
| AH20 | *Levisticum officinale* | MG218304; MG770573; KT944674; KC812809; GU395146. | 100 | A | Anhui | OR030228 |
| AH21 | Unsuccessful amplification | / | / | / | Anhui | / |
| AH22 | *Notopterygium franchetii* | MF096526; MF787580; KY848846; KX674897; JQ936555; JQ936553; MH807979; JF755943; JF694085; GU390407; AY038222. | 100 | G | Anhui | OR030177 |
| AH23 | *Angelica amurensis* | MH188440; MW433735; MN877395; MT735426; JN603227; GU395148; DQ263581. | 100 | A | Anhui | OR030246 |
| AH24 | *Angelica amurensis* | MH188440; MW433735; MN877395; MT735426; JN603227; GU395148; DQ263581. | 100 | A | Anhui | OR030247 |
| AH25 | *Angelica amurensis* | MH188440; MW433735; MN877395; MT735426; MT735425; JN603227; GU395148; DQ263581. | 100 | A | Anhui | OR030248 |
| AH26 | *Levisticum officinale* | MG218304; MG770573; KT944674; KC812809; GU395146. | 100 | A | Anhui | OR030229 |
| CQ01 | *Levisticum officinale* | MG218304; MG770573; KT944674; KC812809; GU395146. | 100 | A | Chongqing | OR030231 |
| CQ02 | Unsuccessful amplification | / | / | / | Chongqing | / |
| CQ03 | Unsuccessful amplification | / | / | / | Chongqing | / |
| CQ04 | Unsuccessful amplification | / | / | / | Chongqing | / |
| CQ05 | Unsuccessful amplification | / | / | / | Chongqing | / |
| CQ06 | Unsuccessful amplification | / | / | / | Chongqing | / |
| CQ07 | Unsuccessful amplification | / | / | / | Chongqing | / |
| CQ08 | Unsuccessful amplification | / | / | / | Chongqing | / |
| CQ09 | Unsuccessful amplification | / | / | / | Chongqing | / |
| CQ10 | Unsuccessful amplification | / | / | / | Chongqing | / |
| CQ11 | *Levisticum officinale* | MG218304; MG770573; KT944674; KC812809; GU395146. | 100 | A | Chongqing | OR030232 |
| CQ12 | *Angelica amurensis* | MH188440; MW433735; MN877395; MT735426; JN603227; GU395148; DQ263581. | 100 | A | Chongqing | OR030249 |
| GD01 | *Notopterygium incisum* | MF096521; MF787531; KY848836; KX674900; MN049521; MT337434; JQ936557; KJ999463; GQ379335; EU236180. | 100 | G | Guangdong | OR030214 |
| GD02 | *Notopterygium incisum* | MF096521; MF787531; KY848836; KX674900; MN049521; MT337434; JQ936557; KJ999463; GQ379335; EU236180. | 100 | G | Guangdong | OR030215 |
| GD03 | *Angelica amurensis* | MH188440; MW433735; MN877395; MT735426; JN603227; GU395148; DQ263581. | 100 | A | Guangdong | OR030253 |
| GD04 | *Notopterygium franchetii* | MF096526; MF787580; KY848846; KX674897; JQ936555; MH807979; JF755943; JF694085. | 100 | G | Guangdong | OR030162 |
| GD05 | *Notopterygium incisum* | MF096521; KY848836; KX674900; MN049521; MT337434; JQ936557; KJ999463; GQ379335; EU236180. | 100 | G | Guangdong | OR030216 |
| GD06 | Unsuccessful amplification | / | / | / | Guangdong | / |
| GD07 | *Notopterygium incisum* | MF096521; MF787531; KY848836; KX674900; KJ999460; MN049521; MT337434; JQ936557; KJ999463; GQ379335; EU236180. | 100 | G | Guangdong | OR030225 |
| GD08 | *Notopterygium franchetii* | MF096522; MF787561; KY848846; KX674897; JQ936555; MH807979; JF755943; JF694085; GU390407; AY038222. | 100 | G | Guangdong | OR030173 |
| GD09 | Unsuccessful amplification | / | / | / | Guangdong | / |
| GS01 | *Notopterygium incisum* | MF096520; MF787527; MF787514; KY848835; KX675120; KX674899; MN049522; MN712237; JF755942; AB558252; DQ278168; MK036626; AY925166; AY038223. | 100 | G | Gansu | OR030191 |
| GS02 | *Angelica amurensis* | MH188440; MW433735; MN877395; MT735426; JN603227; GU395148; DQ263581. | 100 | A | Gansu | OR030250 |
| GS03 | *Levisticum officinale* | MG218304; MG770573; KT944674; KC812809; GU395146. | 100 | A | Gansu | OR030230 |
| GS04 | *Notopterygium franchetii* | MG770576. | 100 | G | Gansu | OR030180 |
| GS05 | *Notopterygium incisum* | MF096521; MF787531; KY848836; KX674900; MN049520; MT337434; JQ936557; KJ999463; GQ379335; EU236180. | 100 | G | Gansu | OR030206 |
| GS06 | Unsuccessful amplification | / | / | / | Gansu | / |
| GS07 | *Angelica amurensis* | MH188440; MW433735; MN877395; MT735426; JN603227; GU395148; DQ263581. | 100 | A | Gansu | OR030255 |
| GS08 | *Notopterygium franchetii* | MF096526; KY848846; KX674897; JQ936555; JQ936553; MH807979; JF755943; JF694085; GU390407; AY038222. | 100 | G | Gansu | OR030178 |
| GS09 | Unsuccessful amplification | / | / | / | Gansu | / |
| GS10 | *Notopterygium incisum* | MF096521; MF787531; KY848836; KX674900; MN049521; MT337434; JQ936557; KJ999463; GQ379335; EU236180. | 100 | G | Gansu | OR030207 |
| GX01 | *Notopterygium incisum* | MF096521; MF787531; MF787511; KY848836; KX674900; MN049521; MT337434; JQ936557; KJ999463; GQ379335; EU236180. | 100 | G | Guangxi | OR030213 |
| GX02 | Unsuccessful amplification | / | / | / | Guangxi | / |
| GX03 | *Ostericum scaberulum* | OK041318; MT735406; DQ270198. | 100 | A | Guangxi | OR030278 |
| GX04 | Unsuccessful amplification | / | / | / | Guangxi | / |
| GX05 | *Notopterygium incisum* | MF096520; MF787527; KY848835; KX675120; KX674899; MN049522; MN712237; JF755942; AB558252; DQ278168; MK036626; AY925166; AY038223. | 100 | G | Guangxi | OR030199 |
| GX06 | *Levisticum officinale* | MG218304; MG770573; KT944674; KC812809; GU395146. | 100 | A | Guangxi | OR030233 |
| GX07 | Unsuccessful amplification | / | / | / | Guangxi | / |
| GX08 | *Notopterygium incisum* | MF096520; MF787527; KY848835; KX675120; KX674899; MN049522; MN712237; JF755942; AB558252; DQ278168; MK036626; AY925166; AY038223. | 100 | G | Guangxi | OR030200 |
| GX09 | Unsuccessful amplification | / | / | / | Guangxi | / |
| GX10 | Unsuccessful amplification | / | / | / | Guangxi | / |
| GX11 | Unsuccessful amplification | / | / | / | Guangxi | / |
| GZ01 | *Notopterygium franchetii* | MF096526; MF787580; KY848846; KX674897; JQ936555; MH807979; JF755943; JF694085. | 100 | G | Guizhou | OR030163 |
| GZ02 | *Notopterygium incisum* | MF096520; MF787527; KY848835; KX675120; KX674899; MN049522; MN712237; JF755942; AB558252; DQ278168; MK036626; AY925166; AY038223. | 100 | G | Guizhou | OR030187 |
| GZ03 | Unsuccessful amplification | / | / | / | Guizhou | / |
| GZ04 | *Notopterygium incisum* | MF096520; MF787527; KY848835; KX675120; KX674899; MN049522; MN712237; JF755942; AB558252; DQ278168; MK036626; AY925166; AY038223. | 100 | G | Guizhou | OR030188 |
| HB01 | *Notopterygium oviforme* | MF787548; KY848837; MT337430. | 100 | A | Hebei | OR030268 |
| HB02 | *Notopterygium incisum* | MF096520; MF787527; KY848835; KX675120; KX674899; MN049522; MN712237; JF755942; AB558252; DQ278168; MK036626; AY925166; AY038223. | 100 | G | Hebei | OR030182 |
| HB03 | *Notopterygium incisum* | MF096520; KY848835; KX675120; KX674899; MN049522; MN712237; JF755942; AB558252; DQ278168; MK036626; AY925166; AY038223. | 100 | G | Hebei | OR030183 |
| HB04 | *Notopterygium franchetii* | MF096526; MF787580; KY848846; KX674897; JQ936555; MH807979; JF755943; JF694085; GU390407; AY038222. | 100 | G | Hebei | OR030168 |
| HB05 | *Notopterygium incisum* | MF096520; MF787527; KY848835; KX675120; KX674899; MN049522; MN712237; JF755942; AB558252; DQ278168; MK036626; AY925166; AY038223. | 100 | G | Hebei | OR030184 |
| HB06 | *Notopterygium franchetii* | MF096522; MF787561; KY848846; KX674897; JQ936555; MH807979; JF755943; JF694085. | 100 | G | Hebei | OR030169 |
| HB07 | *Notopterygium franchetii* | KX675119; JQ936554; JF755944. | 100 | G | Hebei | OR030179 |
| HB08 | *Notopterygium incisum* | MF096520; MF787527; KY848835; KX675120; KX674899; MN049522; MN712237; JF755942; AB558252; DQ278168; MK036626; AY925166; AY038223. | 100 | G | Hebei | OR030185 |
| HB09 | *Notopterygium incisum* | MF096520; MF787527; KY848835; KX675120; KX674899; MN049522; MN712237; JF755942; AB558252; DQ278168; MK036626; AY925166; AY038223. | 100 | G | Hebei | OR030186 |
| HLJ01 | Unsuccessful amplification | / | / | / | Heilongjiang | / |
| HLJ02 | Unsuccessful amplification | / | / | / | Heilongjiang | / |
| HLJ03 | *Notopterygium incisum* | MF096521; KY848836; KX674900; MN049520; MT337434; JQ936557; KJ999463; GQ379335; EU236180. | 100 | G | Heilongjiang | OR030204 |
| HLJ04 | *Notopterygium incisum* | MF096521; MF787531; KY848836; KX674900; MN049520; MT337434; JQ936557; KJ999463; GQ379335; EU236180. | 100 | G | Heilongjiang | OR030203 |
| HLJ05 | Unsuccessful amplification | / | / | / | Heilongjiang | / |
| HLJ06 | Unsuccessful amplification | / | / | / | Heilongjiang | / |
| HLJ07 | *Levisticum officinale* | MG218304; MG770573; KT944674; KC812809; GU395146. | 100 | A | Heilongjiang | OR030234 |
| HLJ08 | Unsuccessful amplification | / | / | / | Heilongjiang | / |
| HJL09 | *Levisticum officinale* | MG218304; MG770573; KT944674; KC812809; GU395146. | 100 | A | Heilongjiang | OR030235 |
| HLJ10 | *Notopterygium incisum* | MF096521; KY848836; KX674900; MN049520; MT337434; JQ936557; KJ999463; GQ379335; EU236180. | 100 | G | Heilongjiang | OR030205 |
| HN01 | *Notopterygium franchetii* | MF096526; MF787580; KY848846; KX674897; JQ936555; JQ936553; MH807979; JF755943; JF694085; GU390407; AY038222. | 100 | G | Hunan | OR030174 |
| HN02 | Unsuccessful amplification | / | / | / | Hunan | / |
| HN03 | Unsuccessful amplification | / | / | / | Hunan | / |
| HN04 | Unsuccessful amplification | / | / | / | Hunan | / |
| HN05 | Unsuccessful amplification | / | / | / | Hunan | / |
| JL01 | *Notopterygium incisum* | MF096520; MF787527; KY848835; KX675120; KX674899; MN049522; MN712237; JF755942; AB558252; DQ278168; MK036626; AY925166; AY038223. | 100 | G | Jilin | OR030189 |
| JL02 | *Notopterygium oviforme* | MF787547; KY848837; MT337430. | 100 | A | Jilin | OR030267 |
| JL03 | Unsuccessful amplification | / | / | / | Jilin | / |
| JS01 | *Notopterygium incisum* | MF096521; MF787531; KY848836; KX674900; MN049521; MT337434; JQ936557; KJ999463; GQ379335; EU236180. | 100 | G | Jiangsu | OR030220 |
| JX01 | *Notopterygium franchetii* | MF096522; MF787565; KY848846; KX674897; JQ936555; MH807979; JF755943; JF694085. | 100 | G | Jiangxi | OR030170 |
| JX02 | *Notopterygium oviforme* | MF787548; KY848837; MT337430. | 100 | A | Jiangxi | OR030266 |
| LL01 | *Levisticum officinale* | MG218304; MG770573; KT944674; KC812809; GU395146. | 100 | A | Shanxi | OR030236 |
| LL02 | Unsuccessful amplification | / | / | / | Shanxi | / |
| LN01 | *Levisticum officinale* | MG218304; MG770573; KT944674; KC812809; GU395146. | 100 | A | Liaoning | OR030237 |
| LN02 | *Levisticum officinale* | MG218304; MG770573; KT944674; KC812809; GU395146. | 100 | A | Liaoning | OR030238 |
| LN03 | *Levisticum officinale* | MG218304; MG770573; KT944674; KC812809; GU395146. | 100 | A | Liaoning | OR030239 |
| LN04 | Unsuccessful amplification | / | / | / | Liaoning | / |
| LN05 | Unsuccessful amplification | / | / | / | Liaoning | / |
| LN06 | *Levisticum officinale* | MG218304; MG770573; KT944674; KC812809; GU395146. | 100 | A | Liaoning | OR030240 |
| NM01 | *Angelica amurensis* | MH188440; MW433735; MN877395; MT735426; JN603227; GU395148; DQ263581. | 100 | A | Neimenggu | OR030251 |
| QC01 | Unsuccessful amplification | / | / | / | Hubei | / |
| QC02 | Unsuccessful amplification | / | / | / | Hubei | / |
| QC03 | Unsuccessful amplification | / | / | / | Hubei | / |
| QC04 | Unsuccessful amplification | / | / | / | Hubei | / |
| QC05 | *Notopterygium oviforme* | MF787548; KY848837; MT337430. | 100 | A | Hubei | OR030270 |
| QC06 | *Notopterygium franchetii* | MF096526; MF787580; KY848846; KX674897; JQ936555; MH807979; JF755943; JF694085; GU390407; AY038222. | 100 | G | Hubei | OR030176 |
| QC07 | *Notopterygium incisum* | MF096521; MF787531; KY848836; KX674900; MN049521; MT337434; JQ936557; KJ999463; GQ379335; EU236180. | 100 | G | Hubei | OR030208 |
| QH01 | *Notopterygium incisum* | MF096520; MF787527; KY848835; KX675120; KX674899; MN049522; MN712237; JF755942; AB558252; DQ278168; MK036626; AY925166; AY038223. | 100 | G | Qinghai | OR030190 |
| SC01 | Unsuccessful amplification | / | / | / | Sichuan | / |
| SC02 | *Notopterygium incisum* | MF096521; MF787531; KY848836; KX674900; KJ999460; MN049521; MT337434; JQ936557; KJ999463; GQ379335; EU236180. | 100 | G | Sichuan | OR030219 |
| SC03 | *Notopterygium incisum* | MF096520; MF096516; MF787527; KY848835; KX675120; KX674899; MN049522; MN712237; JF755942; AB558252; DQ278168; MK036626; AY925166; AY038223. | 100 | G | Sichuan | OR030192 |
| SC04 | *Notopterygium incisum* | MF096520; MF787527; KY848835; KX675120; KX674899; MN049522; MN712237; JF755942; AB558252; DQ278168; MK036626; AY925166; AY038223; | 100 | G | Sichuan | OR030193 |
| SC05 | *Notopterygium incisum* | MF096520; MF787527; KY848835; KX675120; MN049522; MN712237; JF755942; AB558252; DQ278168; MK036626; AY925166; AY038223. | 100 | G | Sichuan | OR030194 |
| SC06 | *Notopterygium incisum* | MF096520; MF787527; KY848835; KX675120; KX674899; MN049522; MN712237; JF755942; AB558252; DQ278168; MK036626; AY925166; AY038223. | 100 | G | Sichuan | OR030195 |
| SC07 | Unsuccessful amplification | / | / | / | Sichuan | / |
| SC08 | *Notopterygium incisum* | MF096520; MF787527; KY848835; KX675120; KX674899; MN049522; MN712237; JF755942; AB558252; DQ278168; MK036626; AY925166; AY038223. | 100 | G | Sichuan | OR030196 |
| SC09 | *Notopterygium incisum* | MF096521; KY848836; KX674900; MN049521; MN049520; MT337434; JQ936557; KJ999463; GQ379335; EU236180. | 100 | G | Sichuan | OR030209 |
| SC10 | Unsuccessful amplification | / | / | / | Sichuan | / |
| SC11 | Unsuccessful amplification | / | / | / | Sichuan | / |
| SC12 | *Notopterygium oviforme* | MF787548; KY848837; MT337430. | 100 | A | Sichuan | OR030262 |
| SC13 | *Levisticum officinale* | MG218304; MG770573; KT944674; KC812809; GU395146. | 100 | A | Sichuan | OR030242 |
| SC14 | Unsuccessful amplification | / | / | / | Sichuan | / |
| SC15 | *Notopterygium oviforme* | MF787548; KY848837; MT337430. | 100 | A | Sichuan | OR030263 |
| SC16 | *Heracleum fargesii* | MF803803; MG220206; MG217824; MG237358; KJ157633; MF803804; HQ686508; FJ812114; EU169278; EU594922; DQ427047; HQ686350; FJ812112; AH003476.2 DQ927298. | 100 | A | Sichuan | OR030279 |
| SD01 | *Notopterygium oviforme* | MF787548; KY848837; MT337430. | 100 | A | Shandong | OR030264 |
| SD02 | *Angelica amurensis* | MH188440; MW433735; MN877395; MT735426; JN603227; GU395148; DQ263581. | 100 | A | Shandong | OR030252 |
| SD03 | *Notopterygium oviforme* | MF787548; KY848837; MT337430. | 100 | A | Shandong | OR030265 |
| SD04 | *Notopterygium incisum* | MF096520; MF787527; KY848835; KX675120; KX674899; MN049522; MN712237; JF755942; AB558252; DQ278168; MK036626; AY925166; AY038223. | 100 | G | Shandong | OR030202 |
| SD05 | *Notopterygium franchetii* | MF096526; MF787580; KY848846; KX674897; JQ936555; MH807979; JF755943; JF694085; GU390407; AY038222. | 100 | G | Shandong | OR030171 |
| SD06 | Unsuccessful amplification | / | / | / | Shandong | / |
| SD07 | Unsuccessful amplification | / | / | / | Shandong | / |
| SX01 | *Notopterygium incisum* | MF096521; MF787531; KY848836; KX674900; MN049521; MT337434; JQ936557; KJ999463; GQ379335; EU236180. | 100 | G | Shaanxi | OR030210 |
| SX02 | *Notopterygium incisum* | MF096520; MF787527; KY848835; KX675120; KX674899; MN049522; MN712237; JF755942; AB558252; DQ278168; MK036626; AY925166; AY038223. | 100 | G | Shaanxi | OR030201 |
| SX03 | Unsuccessful amplification | / | / | / | Shaanxi | / |
| SX04 | *Notopterygium franchetii* | MF096526; MF787580; KY848846; KX674897; JQ936555; MH807979; JF755943; JF694085; GU390407; AY038222. | 100 | G | Shaanxi | OR030175 |
| SX05 | *Levisticum officinale* | MG218304; MG770573; KT944674; KC812809; GU395146. | 100 | A | Shaanxi | OR030241 |
| SX06 | *Notopterygium incisum* | MF096521; MF787531; KY848836; KX674900; MN049521; MT337434; JQ936557; KJ999463; GQ379335; EU236180. | 100 | G | Shaanxi | OR030211 |
| SX07 | *Notopterygium franchetii* | MF096526; MF787580; KY848846; KX674897; JQ936555; MH807979; JF755943; JF694085; GU390407; AY038222. | 100 | G | Shaanxi | OR030181 |
| SX08 | *Notopterygium incisum* | MF096521; MF787531; KY848836; KX674900; MN049521; MT337434; JQ936557; KJ999463; GQ379335; EU236180. | 100 | G | Shaanxi | OR030212 |
| XZ01 | *Haplosphaera himalayensis* | MT337433; MT217048. | 100 | A | Xizang | OR030272 |
| XZ02 | *Haplosphaera himalayensis* | MT337433; MT217048. | 99.56/99.55 | A | Xizang | OR030273 |
| YN01 | *Ostericum scaberulum* | OK041318; MT735406; DQ270198. | 100 | A | Yunnan | OR030274 |
| YN02 | *Notopterygium incisum* | MF096521; MF787531; KY848836; KX674900; KJ999460; MN049521; MT337434; JQ936557; KJ999463; GQ379335; EU236180. | 100 | G | Yunnan | OR030221 |
| YN03 | Unsuccessful amplification | / | / | / | Yunnan | / |
| YN04 | *Notopterygium franchetii* | MF096526; MF787580; KY848846; KX674897; JQ936555; MH807979; JF755943; JF694085; GU390407; AY038222. | 100 | G | Yunnan | OR030172 |
| YN05 | *Notopterygium incisum* | MF096520; MF787527; KY848835; KX675120; KX674899; MN049522; MN712237; JF755942; AB558252; DQ278168; MK036626; AY925166; AY038223. | 100 | G | Yunnan | OR030197 |
| YN06 | *Ostericum scaberulum* | OK041318; MT735406; DQ270198. | 100 | A | Yunnan | OR030275 |
| YN07 | *Ostericum scaberulum* | OK041318; MT735406; DQ270198. | 100 | A | Yunnan | OR030276 |
| YN08 | *Ostericum scaberulum* | OK041318; MT735406; DQ270198. | 100 | A | Yunnan | OR030277 |
| YN09 | *Notopterygium oviforme* | MF787545; KY848838. | 100 | A | Yunnan | OR030269 |
| YN10 | *Notopterygium oviforme* | MF787545; KY848839. | 100 | A | Yunnan | OR030271 |
| YN11 | *Notopterygium incisum* | MF096520; MF787527; KY848835; KX675120; KX674899; MN049522; MN712237; JF755942; AB558252; DQ278168; MK036626; AY925166; AY038223. | 100 | G | Yunnan | OR030198 |
| YN12 | *Notopterygium incisum* | MF096521; MF787531; KY848836; KX674900; KJ999460; MN049521; MT337434; JQ936557; KJ999463; GQ379335; EU236180. | 100 | G | Yunnan | OR030222 |
| YN13 | *Broussonetia papyrifera* | MN381762; MH711090; KT207496; KP096067; MT227730; HM623778; GQ434329; | 100 | A | Yunnan | OR030280 |
| YN14 | *Broussonetia papyrifera* | MN381762; MH711090; KT207496; KP096067; MT227730; HM623778; GQ434329. | 100 | A | Yunnan | OR030281 |
| ZJ01 | *Notopterygium incisum* | MF096521; MF787531; KY848836; KX674900; KJ999460; MN049521; MT337434; JQ936557; KJ999463; GQ379335; EU236180. | 100 | G | Zhejiang | OR030223 |
| ZJ02 | *Notopterygium incisum* | MF096521; MF787531; KY848836; KX674900; KJ999460; MN049521; MT337434; JQ936557; KJ999463; GQ379335; EU236180. | 100 | G | Zhejiang | OR030224 |

Note: G-genuine; A-adulterant

| **Table S2**. Overview of the NReR sold scenario on medicinal markets in China. | | | |
| --- | --- | --- | --- |
| Province | NReR | Adulterant | Failure*^a^* |
| Anhui | 8 | 16 | 2 |
| Chongqing | 0 | 3 | 9 |
| Gansu | 5 | 3 | 2 |
| Guangdong | 6 | 1 | 2 |
| Guangxi | 3 | 2 | 6 |
| Guizhou | 3 | 0 | 1 |
| Hebei | 8 | 1 | 0 |
| Heilongjiang | 3 | 2 | 5 |
| Hubei | 2 | 1 | 4 |
| Hunan | 1 | 0 | 4 |
| Jiangsu | 1 | 0 | 0 |
| Jiangxi | 1 | 1 | 0 |
| Jilin | 1 | 1 | 1 |
| Liaoning | 0 | 4 | 2 |
| Neimenggu | 0 | 1 | 0 |
| Qinghai | 1 | 0 | 0 |
| Shaanxi | 6 | 1 | 1 |
| Shandong | 2 | 3 | 2 |
| Shanxi | 0 | 1 | 1 |
| Sichuan | 7 | 4 | 5 |
| Xizang | 0 | 2 | 0 |
| Yunnan | 5 | 8 | 1 |
| Zhejiang | 2 | 0 | 0 |

*a*: The number of samples with failed PCR amplification.
